# Poisoning from Medicine Prescribed by Mistake

**Published:** 1829-10-01

**Authors:** 


XLIX.

### Poisoning from Medicine prescribed by mistake.

Two very melancholy instances of poisoning have lately occurred; the first in France, the latter in this country. If they were not expressly calculated to teach caution, where caution is pre-eminently required, we should not think it necessary to notice them in these pages, for a newspaper is the proper vehicle for the ordinary run of shocking occurrences. We have no peculiar taste for mere horrors ourselves, nor any desire to minister to such a penchant in the public, but when we can convey a salutary lesson it becomes an unpleasant duty to do so.

It seems, then, that on the 26th of May, Dr. Buet, of Paris, was summoned in the evening to attend a little girl, ætatis five, child of a M. Gohyer, who laboured under fever of an intermittent type. Dr. Buet determined to prescribe the *sulphate of quinine*, and as the stomach was irritable he thought it better to give it in the form of glyster. He accordingly wrote the following prescription.

"℞. *Sulphate de Morphine*, gr. x.

"*The apothecary is to add two or three drops of sulphuric acid, in order to render the sub-sulphate soluble in a glass of water, which is to be given as a glyster to the child.*"

The above prescription was given to Madame Gohyer, without Dr. Buet's perceiving that he had written *morphine* instead of *quinine*, and it was sent by the lady to the apothecary. He returned it to ascertain whether the quantity of the sulphate of morphine was correctly stated, and the mother assured him that it was, having heard Dr. Buet mention ten grains. All doubts were removed, and the fatal enema administered to the child. In the course of ten minutes it began to sleep, and shortly afterwards it was seized with such alarming convulsions, that Dr. Buet was hastily summoned. The error was now discovered, and the scene that ensued would seem to have beggared description. Other medical men were summoned, and purgative glysters, with vinegar and water, were administered or tried to be administered in the interim. When all had collected, purgative enemas with coffee and vinegar, &c. were had recourse to, but without effect, and at 9, p. m. the little patient expired.

M. Buet, who communicates this melancholy case to the Editor of the *Clinique*, concludes by observing, that he leaves to his brethren the task of condemning him for the sad mistake of a word which he made in the prescription, a mistake which can only be laid to the weakness, or rather the *vertigo* of the human mind. Alas! the anguish and the punishment, both moral and personal, of such mistakes are severe enough, God knows, without the gall and wormwood of professional crimination! We would merely remark, that no prescription should ever be committed to the hands of the chemist or assistant before it has been carefully read over. This simple precaution might prevent a great portion of those errors that are constantly committed, though not on such a scale as to peril life.

The next case which occurred at Finchley and was made the subject of a coroner's inquest, is fully related in the Times newspaper, for September the 9th. We shall not detail the particulars but merely glance at the leading facts.

It seems that a Mrs. Phillips of Finchley, married only five months, sent for Mr. Snow, an apothecary at Highgate, on Sunday Sept. 6th. Mr. Snow being in Hertfordshire, Dr. Tweedie of Ely Place, Holborn, visited the patient for him, and found her suffering from disordered (*disorganized* is the newspaper version) bowels and stomach with nervous irritability and flightiness. On Dr. Tweedie's return to Mr. Snow's he prescribed some medicine for the lady, which was compounded and sent by Mr. Hill, the assistant. On Thursday Dr. Tweedie called again upon the patient, and found reason to alter the prescription, which then ran as follows:—

℞. *Submur. hydr.* grs. ij. *Ext. coloc. co.* gr. viij. *Olei carui.* gtt. ij. *Contunde et fiant pilulæ duæ, h. s. s.*

℞. *Infus. cascar.* ℥ij. *Infusi sennæ*, ℥iij. *Mannæ*, ℥ss. *Tinct. gentian, co.* ℥j. *Misce. Sit mistura, cujus capiat coch. tria ampla ter quotidie.*

*For Mrs. Phillips, 3d Sept.* 1829. *A. T.*

The mixture from the assistant purporting to be the above, was received by Mrs. Phillips on Thursday evening, and early on Friday morning, when her husband left her, she appeared quite well. On his return, however, about 11 o'clock at night, he was informed that she had been very unwell, and being oppressed with drowsiness had retired to her chamber some time. Another surgeon, Mr. Hammond, had been sent for, but he also was absent and his assistant came, who, on seeing Mrs. Phillips, observed that the drowsiness was produced by the mixture she had taken, and that if not disturbed she would be better in the morning. The husband accordingly retired to rest without awaking her, but when the morning appeared he found her a corpse by his side! Various medical men were summoned in haste, and Mr. Hammond, who examined the mixture, said that it was chiefly composed of laudanum. Dr. Tweedie too, on his arrival, declared on tasting it that it was not composed according to his prescription, which has been shewn already to have contained no laudanum whatever.

On examining the body, the brain was found to be more vascular than usual; the blood-vessels of the membranes were turgid with blood, and presented a slight milky effusion—the thoracic viscera were sound—the coats of the stomach were inflamed in patches—the intestines were perfectly healthy. From this *post-mortem* examination Dr. Tweedie gave in evidence his opinion, that the deceased's death was occasioned by her having taken an overdose of laudanum.

Mr. Swan Hill, the assistant, swore that he had made up Dr. Tweedie's two prescriptions—that there was no laudanum in either of them—that they were sent with proper directions—that he made up *no other* prescription on the Thursday, and that there were *no* bottles of laudanum on the shelves in the shop. This gentleman is 25 years of age, has served five years' apprenticeship to a chemist, and has been in the habit of compounding medicines since his apprenticeship, which is upwards of three years.

The inquest lasted ten hours, and the verdict returned by the Jury was, "that the deceased's death was occasioned by an over-dose of laudanum taken medicinally."

Without attempting to fasten blame on any individual for this melancholy catastrophe, we cannot but remark that the supposition of the deceased having taken laudanum as a poison, appears to us to be next to incredible. The poor lady had only been five months married, was by all accounts living happily with an affectionate husband, and was even proved to be particularly solicitous about her health. The production, too, of Dr. Tweedie's prescription exculpates this talented and amiable physician most completely from the charge of error or negligence. But how then did this fatal mistake, if such it was, occur? Could any thing have been employed instead of the compound tincture of gentian?* God knows that the naked suspicion of such a circumstance is a fearful cloud on the mind and the prospects of a medical man!

We have been induced to give these cases in the hope that they may serve as a useful lesson to practitioners. We tremble to think how often the life of individuals hangs by the brittle thread of an ignorant apprentice or assistant's circumspection.

---

### L.

### REGULATIONS TO BE OBSERVED BY CANDIDATES FOR DIPLOMAS FROM THE ROYAL COLLEGE OF SURGEONS OF EDINBURGH.

THE present is decidedly the age of me-

---

* Mr. Hill has since published a letter in the newspapers, which proves that he *could not* have committed this or any other mistake. We communicate this fact with the utmost pleasure.—*Rev.*

